# Letter to the editor: comments on “prognostic significance of lactate/albumin ratio in respiratory failure and sepsis”

**DOI:** 10.1080/07853890.2026.2627132

**Published:** 2026-02-10

**Authors:** Yuru Fu, Yuanyuan Wang

**Affiliations:** ^a^ Department of Intensive Care Unit, Fuyang People’s Hospital of Anhui Medical University, Fuyang, China; ^b^ Department of Cardiology, Fuyang People’s Hospital of Anhui Medical University, Fuyang, China

**Keywords:** Sepsis, acute respiratory failure, lactate/albumin ratio, methodological critique

Acharya et al. recently published a prospective cohort study in Annals of Medicine investigating the prognostic value of the lactate/albumin ratio (LAR) in patients with sepsis, acute respiratory failure (ARF), or both [1]. The authors reported that LAR exhibited superior predictive performance compared to the Sequential Organ Failure Assessment (SOFA) score and albumin alone for in-hospital mortality, need for mechanical ventilation, and inotrope use, while being comparable to lactate alone. Although these findings contribute to the growing evidence supporting LAR as a bedside prognostic marker [2], several methodological and interpretive issues warrant discussion.

Primarily, the use of DeLong’s test to compare receiver operating characteristic (ROC) area under the curve (AUC) values is appropriate for assessing differences between LAR and comparators like lactate or SOFA. However, many comparisons yielded non-significant p-values (e.g. *p* = 0.24 for LAR vs. lactate in predicting mortality in sepsis). These results suggest statistical equivalence rather than superiority – a nuance that may have been overstated. In smaller subgroups, such as the combined sepsis and ARF cohort (*n* = 43), limited statistical power may lead to type II errors. A post hoc power analysis could clarify this, as seen in similar MIMIC-IV database studies where sample sizes below 100 reduced the reliability of AUC comparisons [3].

Additionally, reliance on Youden’s index-which maximizes (sensitivity + specificity − 1) – to determine optimal cutoffs (e.g. 1.78 for mortality in sepsis) may not align with clinical scenarios that prioritize sensitivity in life-threatening conditions. Alternative approaches, such as cost-sensitive thresholds or decision curve analysis, could provide more actionable insights, especially given the variability in cutoffs across subgroups (which increase with disease severity) [4]. The lack of adjustment for multiple comparisons across numerous ROC analyses (three outcomes per group) also raises concerns about type I error inflation, although false discovery rate correction could mitigate this in future studies. Furthermore, the prognostic role of LAR may be limited when dynamic trends in lactate clearance are considered, which could provide superior insights into resolution or persistence of sepsis compared to a single ratio incorporating albumin levels that decline later in the disease course.

Regarding clinical implications, caution is warranted. While LAR’s prognostic value in ARF is novel, the predominantly Nepalese cohort limits generalizability to diverse populations, where ethnic differences in albumin metabolism could influence ratios [2]. Moreover, integrating LAR with established scores such as Acute Physiology and Chronic Health Evaluation II (APACHE-II), rather than comparing solely to SOFA, might better position it as a complementary tool [5].

In conclusion, Acharya et al.’s work advances LAR as a simple bedside marker, but addressing these statistical nuances could refine its validation. We commend the authors and look forward to further prospective multicenter studies.

## Data Availability

No new data were generated during the study.
